# Chronic kidney disease incidences in Thai outpatients diagnosed with psychiatric illnesses receiving lithium maintenance therapy: a university hospital-based study

**DOI:** 10.1186/s12888-024-05550-4

**Published:** 2024-01-30

**Authors:** Jarurin Pitanupong, Chavisa Jittpratoom, Kanthee Anantapong

**Affiliations:** Department of Psychiatry, Faculty of Medicine, Prince of Songkla University, Hat Yai, Songkhla 90110 Thailand

**Keywords:** Chronic kidney disease, Lithium maintenance therapy, Psychiatric outpatient

## Abstract

**Background:**

There has been no previous study in Thailand regarding the incidence of lithium-induced abnormal renal function. Hence, this study aimed to assess the effect of lithium maintenance therapy on chronic kidney disease, and associated factors among outpatients diagnosed with a psychiatric illness within Southern Thailand.

**Methods:**

This was a retrospective study, using an information review from the electronic medical records of Songklanagarind Hospital computer system in the last ten years; from 1 January 2013 until 31 September 2022. Chronic kidney disease was defined as an estimated glomerular filtration rate of less than 60 mL/min/1.73 m^2^ and persisted for three months or more. There were 461 outpatients diagnosed with a psychiatric illness who received lithium maintenance therapy. From this, 154 outpatients were excluded: 153 received lithium therapy for less than three months and 1 presented with a baseline chronic kidney disease. All data were analyzed using Rstudio 4.3.1. The incidence of lithium-induced chronic kidney disease was analyzed by survival analysis.

**Results:**

Of the 307 outpatients diagnosed with a psychiatric illness and received lithium maintenance therapy, the most common diagnosis was bipolar disorder (59.3%). Most were female (52.8%), with the median (IQR) age of 39.0 (27.5–54.0) years. The median (IQR) age onset of lithium therapy and duration of lithium maintenance therapy were 28.0 (21.0–41.5) years, and 2.97 (0.9–9.2) years, respectively. This study identified six outpatients (1.9%) that developed chronic kidney disease stage 3 or more and one of them (0.3%) presented with chronic kidney disease stage 5 or end-stage. The incidence of lithium-induced chronic kidney disease was 0.0023 cases per exposed patient-year. When comparing outpatients who had received lithium maintenance therapy and developed chronic kidney disease with those who did not develop chronic kidney disease, this study identified that most of the group with chronic kidney disease had a lithium maintenance therapy for more than ten years, had an older age onset of lithium therapy, reported history of psychiatric hospitalization and lithium intoxication, and presented with physical illness. The associated factors between the effect of lithium maintenance therapy and chronic kidney disease could not be identified due to a limited number of outpatients having developed chronic kidney disease.

**Conclusions:**

Lithium-induced chronic kidney disease was identified as a minor incidence, and it was likely safe for maintenance therapy with careful and regular monitoring. However, older patients or those receiving lithium for a longer time and present with comorbid physical illnesses should be prescribed with caution.

**IRB / IEC certification:**

65-389-3-4.

## Background

Over the past decades, lithium has been used as a gold standard therapy for the treatment of bipolar disorders; either in the acute or maintenance phases. Moreover, lithium has been used for anti-suicidal effects [1]. Despite its effectiveness, lithium has been of concern due to its narrow therapeutic index, and its many side effects; such as renal impairment [2, 3]. Therefore, the detection of renal functions, or evaluation of the progression of renal impairment as well as serum levels of lithium should continuously be monitored [4].

The various degrees of glomerular dysfunction are described by terms; such as ‘renal impairment’, ‘renal insufficiency’, and ‘chronic renal failure’. The National Institute for Health and Care Excellence (NICE) clinical guidelines replace such terms with a single category of chronic kidney disease (CKD), which divides this into five stages of severity [5]. If an estimated glomerular filtration rate (eGFR) is less than 60 mL/min/1.73 m^2^ and persists for three months or more, or the presence of albuminuria combined with other markers of kidney deterioration is detected then it is defined as CKD stage 3. CKD is focused on stage 3 due to its association with other major diseases and increased morbidity and mortality rates [6].

CKD is common in both the Thai and worldwide populations. Among population-based studies, the prevalence of CKD in Asia, across 16 countries, has been reported to range from 7.0 to 34.3%. Thailand population-based studies have reported the prevalence of CKD of 10.0 to 14.8% [7]. Moreover, comorbidities; such as essential hypertension, diabetes mellitus, and cardiovascular disease, have been associated with CKD in worldwide populations [6].

Regarding long-term lithium therapy, this has also been associated with renal side effects; such as CKD, and nephrogenic diabetes insipidus [8]. The prevalence of impaired kidney function in long-term lithium therapy is 25.5% [6], and CKD may develop within 5 years after starting lithium therapy [1]. Additionally, several factors, such as duration of lithium used, cumulative lithium dose, prior episodes of lithium intoxication [9], demographic factors of each patient and their comorbidities, and nephrotoxic medication used [10] can impact the rates and severity of impaired kidney function. However, the prior study reported a variable duration of developing CKD among outpatients receiving lithium. Moreover, some studies show no increased risk of renal impairment after adjusting for confounding factors; such as comorbidities or nephrotoxic medications [11].

Over the last decade, accruing evidence regarding the benefits of lithium has led to a resurgence of interest in its clinical use [5]. Although knowledge concerning the association between lithium and chronic impairment in glomerular function has long been debated it is still far from satisfactory. However, this limited evidence does provide a basis for the monitoring and management of a range of renal pathologies. As there has been no study in Thailand regarding the incidence of lithium-induced CKD, this study aimed to address these points. It also aimed to identify determinants; including monitoring and management of renal adverse effects, that could minimize the side-effect burden and optimize the use of lithium.

## Methods

After being approved by the Ethics Committees of the Faculty of Medicine, Prince of Songkla University (REC.65-389-3-4), this retrospective study was conducted using the information reviews from medical records by the hospital computer system of Songklanagarind Hospital; which is an 800-bed university hospital serving as a tertiary center. All outpatients diagnosed with a psychiatric illness in the Department of Psychiatry, Songklanagarind Hospital receiving lithium as maintenance therapy in the last ten years; from the periods: 1 January 2013 until 31 September 2022, were included. The inclusion criteria consisted of outpatients diagnosed with a psychiatric illness; for example, bipolar disorder, schizoaffective disorder, being prescribed lithium as a maintenance therapy for more than three months within the study period. The exclusion criteria were those having a baseline eGFR of less than 60 mL/min/1.73 m^2^, which had persisted for three months or more as being CKD stage 3 before the study and having a prior, established diagnosis of any CKD.

### Sample size calculation

Based on the electronic medical records, there were 461 outpatients diagnosed with a psychiatric illness receiving lithium treatment during the study period. Regarding CKD, using the order “N for survey” for sample size calculation, *P* = 0.25 was used (p from a systematic review and meta-analysis of the prevalence of impaired kidney function in outpatients with long-term lithium maintenance therapy [6]), d = 0.05. When adjusting N with the order: “finite population correction”, the required N would be at least 178 patients. However, it was considered achievable to review the medical records of all outpatients who were diagnosed with a psychiatric illness and received lithium, and this would then maximize its power when performing statistical analyses.

### Data collection

We reviewed the electronic medical records over the intended timeframe, and the following data were collected and used for further analysis.

1. Personal and general demographic information inquired around areas related to gender, age, marital status, religion, level of education, occupation, body weight, physical illness, nephrotoxic medications, and the presence of acute kidney injury.
2. Information on psychiatric illness and treatment inquired around areas related to diagnosis of psychiatric illness, history of hospitalization, profile of lithium therapy, age onset and duration of usage, lithium compliance, side effects, and prior history of intoxication.
3. Laboratory investigations inquired around areas related to serum levels of lithium, serum creatinine, and levels of eGFR,

Using a retrospective chart review, we used the lithium dosage on the day the outpatients met the diagnostic criteria of CKD (for those with CKD) and on the last visit (for those without CKD). Serum lithium levels close to the day of diagnosis of CKD were used for outpatients with lithium-induced CKD, and the last readings of serum lithium levels were used for those without CKD. For levels of eGFR, we used the readings on the day the outpatients met the diagnostic criteria of CKD (for those with CKD) or the last readings in the study period (for those without CKD).

In this study, we defined lithium-induced CKD (a main dependent variable, case = 0 or 1) as having a history of lithium exposure as a maintenance therapy for more than three months and having an eGFR of less than 60 mL/min/1.73 m^2^ and persists for three months or more (CKD stage 3). The patients might be followed up for lithium and eGFR by other professionals; therefore, for the data analysis, we retrieved and included the lithium and eGFR levels from every visit across different clinics over the study timeframe. If the patients were not monitored for renal functions, including serum creatinine and eGFR, after starting lithium, we assumed and reported that they did not develop CKD (case = 0).

Although we did not have a systemic record of lithium compliance of our outpatients, in this study, we scrutinized the clinical notes made by psychiatrists regarding their patients’ lithium compliance. If there was a comment on poor lithium compliance in the notes, the outpatients were classified into a poor compliance group. Because every outpatient in this study continuously visited our clinic, we assumed that outpatients without the comment on poor lithium compliance in the notes were likely to take the medication regularly and, therefore, were considered to have good lithium compliance.

### Data analysis

All data were analyzed to describe the patient demographics, profile of lithium therapy, and medications affecting renal function using descriptive statistics. The results were reported using frequency, proportions, means (standard deviation, SD), and median (interquartile range, IQR). Rstudio 4.3.1 (The R Foundation for Statistical Computing) was used to analyze the data. The incidence of lithium-induced CKD was analyzed by survival analysis.

## Results

### Demographic characteristics

**Fig. 1:**
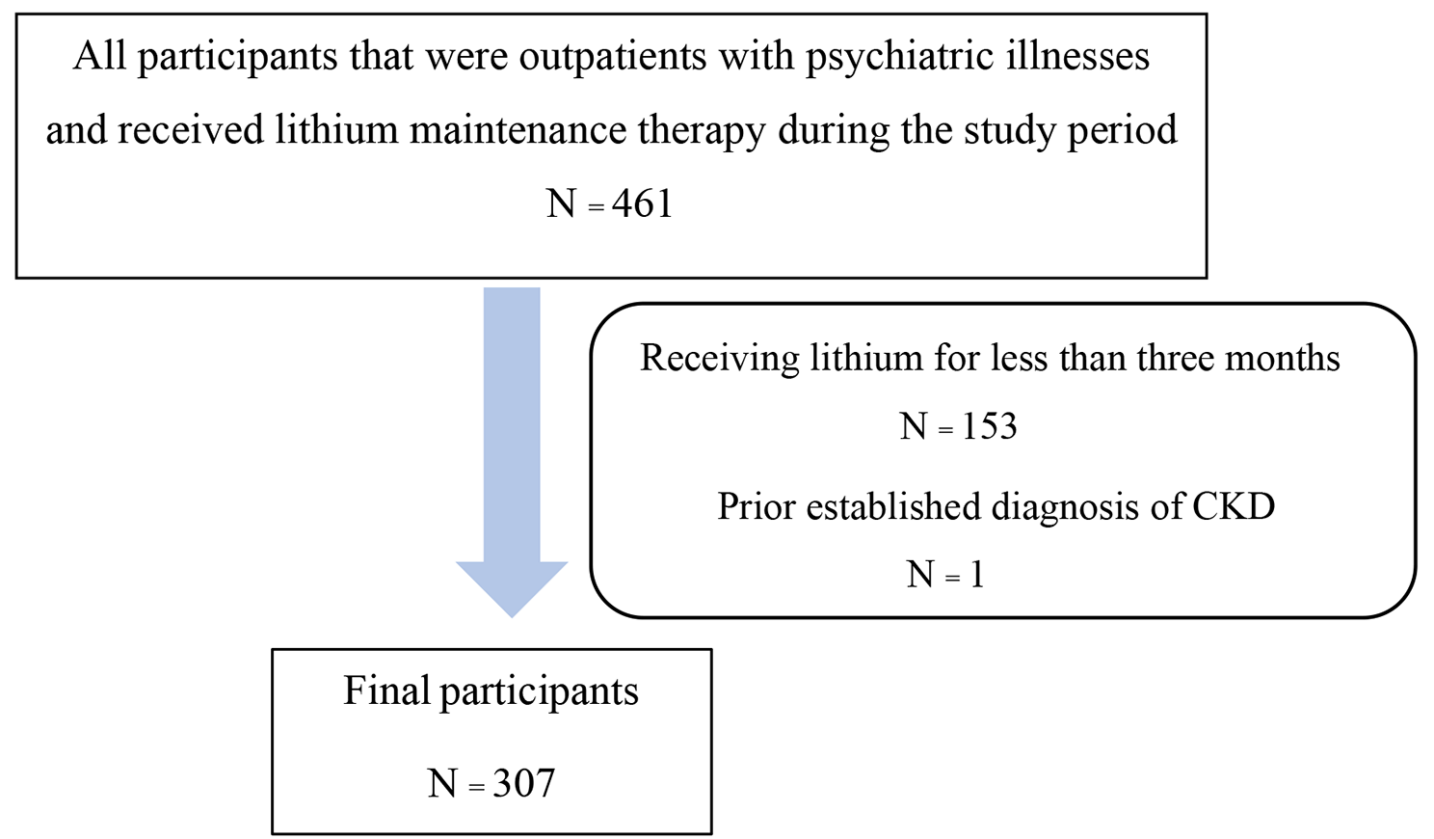
Number of participants

From 461 outpatients diagnosed with a psychiatric illness having received lithium as a maintenance therapy in the last ten years, 154 outpatients were excluded (Fig. 1). Therefore, 307 outpatients were analyzed. They were mostly female (52.8%), with a median (IQR) age and body weight of 39.0 (27.5–54.0) years and 61.0 (51.2–72.0) kilograms, respectively. We found that 77 (25.1%) outpatients had underlying physical illnesses. The most common physical illness was dyslipidemia (13.7%). In addition, 68 (22.1%) outpatients received nephrotoxic medications; for example, Non-Steroidal Anti-Inflammatory Drugs (NSAIDs) (13.4%), and proton pump inhibitors (12.7%), with 3 (1.0%) outpatients developing acute kidney injury (Table 1).

**Table 1:** Demographic characteristics (*N* = 307)

Demographic characteristics	Number (%)
**Gender**	
Male	145 (47.2)
Female	162 (52.8)
**Age** (year)	
≤ 20	9 (2.9)
21–30	100 (32.6)
31–40	59 (19.2)
41–50	47 (15.3)
51–60	43 (14.0)
> 60	49 (16.0)
**Marital status**	
Single	213 (69.4)
Married	83 (27.0)
Divorced/widowed/separated	11 (3.6)
**Religion**	
Buddhism	258 (84.0)
Islam	25 (8.1)
Christianity/other	5 (1.6)
**Level of education**	
Primary school or below	20 (6.5)
Secondary school	7 (2.3)
High school/diploma	27 (8.8)
Bachelor’s degree or above	113 (36.8)
Not specify	140 (45.6)
**Occupation**	
Government officer/state enterprise employee/ company employee	42 (13.7)
Self-employed/merchant/personal business owner/ agriculture	78 (25.4)
Student	103 (33.6)
Unemployed	67 (21.8)
Not specified	17 (5.5)
**Body weight** (kilogram)	
≤ 50	66 (21.5)
51–60	81 (26.4)
61–70	73 (23.8)
71–80	43 (14.0)
> 80	43 (14.0)
Not specify	1 (0.3)
**Presence of physical illness**	
No	230 (74.9)
Yes	77 (25.1)
Dyslipidemia	42 (13.7)
Hypertension	26 (8.5)
Respiratory disease	25 (8.1)
Diabetes Mellitus	20 (6.5)
Liver disease	4 (1.3)
Cardiovascular disorder	3 (1.0)
Immunodeficiency	1 (0.3)
**Receiving nephrotoxic medication**	
No	239 (77.9)
Yes	68 (22.1)
Non-Steroidal Antiinflammatory drugs	41 (13.4)
Proton pump inhibitors Antihypertensive drugs (Angiotensin-converting enzyme inhibitors, Angiotensin receptor blockers, diuretic drugs)	39 (12.7) 16 (5.2)
Amiodarone	1 (0.3)
Antibiotics (Aminoglycoside,Vancomycin)	1 (0.3)
**Presence of acute kidney injury**	
No	304 (99.0)
Yes (Enalapril, and unknown cause)	3 (1.0)

### Characteristics of outpatients with psychiatric illnesses and lithium therapy

Of all 307 outpatients diagnosed with a psychiatric illness, the highest illness diagnosed was bipolar disorder (59.3%) (Table 2). Most outpatients were found to have good lithium compliance (74.3%), with the median (IQR) age of onset for lithium therapy being 28.0 (21.0–41.5) years. The median (IQR) duration of lithium maintenance therapy was 2.97 (0.9–9.2) years. There were 67 (21.8%) outpatients who showed the presence of side effects from lithium; for example, tremors (11.1%), nausea, and vomiting (6.5%). This study found that six outpatients (1.9%) developed CKD, and only one outpatient (0.3%) reported CKD stage 5 or end-stage renal disease. The incidence of lithium-induced CKD was 0.0023 cases per exposed patient-year.

**Table 2:** Characteristic of patients with psychiatric illnesses and lithium therapy (*N* = 307)

Characteristics	Number (%)
**Diagnosis**	
Bipolar disorder	182 (59.3)
Schizoaffective disorder	33 (10.7)
Major depressive disorder	44 (14.3)
Cyclothymia	13 (4.2)
Neurodevelopmental disorder (ADHD, Autism, intellectual disability)	32 (10.4)
Other	3 (9.8)
**History of hospitalization**	
No	155 (50.5)
Yes	152 (49.5)
Both psychiatric illness and physical illness	38 (12.4)
Psychiatric illness	78 (25.4)
Physical illness	36 (11.7)
**Lithium compliance**	
Good	228 (74.3)
Poor	79 (25.7)
**Age onset of lithium therapy** (year) ≤ 20 21–30 31–40 41–50 51–60 > 60	69 (22.5) 99 (32.2) 57 (18.6) 41 (13.4) 29 (9.4) 12 (3.9)
**Dose of lithium usage** (milligram/day)	
300	70 (22.8)
600	140 (45.6)
900	78 (25.4)
1200	16 (5.2)
1500	3 (1.0)
**Serum level of lithium** (mEq/L) < 0.6 0.6–1.2 1.3–1.5 > 1.5 Not available	82 (26.7) 60 (19.5) 6 (1.9) 5 (1.6) 154 (50.2)
**Duration of lithium therapy** (year)	
< 1	86 (28.0)
1–5	107 (34.9)
6–10	45 (14.7)
> 10	69 (22.5)
**Presence of lithium side effects**	
No	240 (78.2)
Yes	67 (21.8)
Tremor	34 (11.1)
Nausea, vomiting	20 (6.5)
Dizziness	13 (4.2)
Dysarthria	3 (1.0)
CKD	6 (1.9)
Stage 3a	5 (1.6)
Stage 3b	0 (0.0)
Stage 4	0 (0.0)
Stage 5	1 (0.3)
**Presence of lithium intoxication**	
No	299 (97.4)
Yes	8 (2.6)

Despite an annual check-up of lithium and renal function levels, including eGFR, being recommended in our hospital, many psychiatrists did not follow this recommendation strictly. There were 98 patients who were not monitored for eGFR after starting lithium; 58 of these were not even assessed for eGFR before commencing lithium therapy. For those who were followed up for eGFR levels after starting lithium, the monitoring was done either by psychiatrists or other professionals for other health conditions. The median interval between each eGFR assessment was 7.5 months (mean = 10.4, SD = 9.0 months) (data not shown in tables). Surprisingly, for lithium levels, 154 patients receiving lithium were not monitored for lithium levels during the study timeframe (Table 2 for ‘Not available’ of serum level of lithium), and one of these patients developed CKD.

### Characteristic of outpatients with lithium-induced chronic kidney disease and non-chronic kidney disease

From Table 3, when comparing the six outpatients who received lithium maintenance therapy and developed CKD with those who did not develop CKD, most of them had had lithium maintenance therapy for over ten years, had an older age onset of lithium therapy, and reported a history of psychiatric hospitalization. In the CKD group, the median age onset of lithium maintenance therapy was 45.5 (IQR = 44.0–51.5) years of age, and the median duration of lithium maintenance therapy being 12.5 (IQR = 6.8–14.8) years. Five of them also presented with a physical illness. The most common physical illnesses among them were hypertension (66.7%), followed by dyslipidemia (33.3%), and diabetes mellitus (33.3%). However, this study could not identify the factors associated with lithium-induced CKD, due to a limited number of outpatients that developed CKD.

**Table 3:** Characteristic of patients with lithium-induced chronic kidney disease and non-chronic kidney disease

Characteristics of patients		Number (%)	
		CKD *N* = 6	Non-CKD *N* = 301
**Diagnosis**			
Bipolar disorder		4 (66.7)	178 (59.1)
Schizoaffective disorder		1 (16.7)	32 (10.6)
Cyclothymia		1 (16.7)	12 (4.0)
Other		0 (0.0)	79 (26.2)
**History of psychiatric hospitalization**			
No		1 (16.7)	195 (64.7)
Yes		5 (83.3)	106 (35.2)
**Age onset of lithium therapy** (year)			
≤ 20		0 (0.0)	69 (22.9)
21–30		1 (16.7)	98 (32.6)
31–40		0 (0.0)	57 (18.9)
41–50		3 (50.0)	38 (12.6)
51–60		2 (33.3)	27 (9.0)
> 60		0 (0.0)	12 (4.0)
Median (IQR)		45.5 (44.0–51.5)	28 (21.0–41.0)
**Dose of lithium usage** (milligram/day)			
300		2 (33.3)	68 (22.6)
600		1 (16.7)	139 (46.2)
900		3 (50.0)	75 (24.9)
1200		0 (0.0)	16 (5.3)
1500		0 (0.0)	3 (1.0)
**Serum level of lithium** (mEq/L)			
< 0.6		0 (0.0)	82 (27.2)
0.6–1.2		2 (33.3)	58 (19.3)
1.3–1.5		1 (16.7)	5 (16.6)
> 1.5		2 (33.3)	3 (1.0)
Not available		1 (16.7)	153 (50.8)
**Duration of lithium therapy** (year)			
< 1		0 (0.0)	86 (28.6)
1–5		1 (16.7)	106 (35.2)
6–10		1 (16.7)	44 (14.6)
> 10		4 (66.7)	65 (21.6)
Median (IQR)		12.5 (6.8–14.8)	2.9 (0.9–8.4)
**Presence of lithium side effects**			
Tremor		3 (50.0)	31 (10.2)
Nausea / vomiting		0 (0.0)	20 (6.6)
Dysarthria		1(16.7)	2 (0.6)
Other		0 (0.0)	13 (4.3)
**Presence of lithium intoxication**			
No		4 (66.7)	295 (98.0)
Yes		2 (33.3)	6 (2.0)
**Receiving nephrotoxic medication**			
No		6 (100.0)	233 (77.4)
Yes		0 (0.0)	68 (22.6)
**Presence of physical illness**			
No		1 (16.7)	228 (75.7)
Yes	5 (83.3)		73 (24.3)

From Table 4, among the six outpatients diagnosed with a psychiatric illness having received lithium maintenance therapy and developing CKD, the proportion of males and females were equal. The dosage of lithium on the day of the diagnosis of CKD ranged from 300 to 900 mg/day. Two out of six outpatients had a history of lithium intoxication. All outpatients did not have a history of acute kidney injury and the use of nephrotoxic medications.

**Table 4:** Characteristic of patients with lithium-induced chronic kidney disease (*N* = 6)

Case	1	2	3	4	5	6
**Gender**	Female	Female	Male	Male	Female	Male
**Psychiatric diagnosis**	Schizoaffective	Bipolar	Cyclothymia	Bipolar	Bipolar	Bipolar
**Age when starting lithium (years)**	44	44	60	27	47	53
**Age at the end of the study (years)**	61	65	64	48	67	65
**Body weight (kg)**	54.1	62	81	86.9	67.2	79.7
**Physical illness**	No	DM, HT	HT	DLP, DM, HT	DLP	HT
**Receiving Nephrotoxic medication**	No	No	No	No	No	No
**Presence of acute kidney injury**	No	No	No	No	No	No
**Lithium dosage at the diagnosis of CKD (mg/d)**	900	300	900	600	600	900
**Lithium compliance**	Poor	Poor	Good	Poor	Good	Good
**Duration using lithium (years)**	12	15	3	18	15	5
**Presence of lithium intoxication**	Yes	No	No	No	No	Yes
**Presence of lithium side effect**	Tremor Dysarthria	Tremor	No	Tremor	No	No
**Serum lithium level (mEq/L)**	1.54	0.81	No data	1.23	0.6	1.59
**CKD stage**	3a	3a	5	3a	3a	3a

**Abbreviations**: CKD = chronic kidney disease, DLP = dyslipidemia, DM = diabetes mellitus, HT = hypertension

## Discussion

This retrospective study was performed to address the concerns regarding lithium in maintenance therapy, and to the best of our knowledge, this is the first study investigating the incidence of lithium-induced abnormal renal function, or CKD, in Thailand. It was found that among the 307 outpatients receiving lithium maintenance therapy, six outpatients (1.9%) developed CKD, with only one outpatient (0.3%) reporting CKD stage 5 or end-stage renal disease. The incidence of lithium-induced CKD was 0.0023 cases per exposed patient-year. In addition, most of the outpatients receiving lithium maintenance therapy who developed CKD had a duration of lithium therapy of over ten years, had an older age onset of lithium therapy, reported a history of psychiatric hospitalization and lithium intoxication, and had a comorbid physical illness. However, this study could not identify the factors associated with lithium-induced CKD, due to a limited number of outpatients developing CKD.

Regarding lithium maintenance therapy and the development of CKD, this study identified that CKD seems to be found in a minority of outpatients. These findings were consistent with a prior study that focused on the incidence of CKD in patients using lithium as maintenance therapy in the Netherlands. The prior incidence of lithium-induced CKD was 0.012 cases per exposed patient year, whereas the incidence of CKD stage 4 was only 0.0004 per patient- year, and no cases of end-stage renal disease were found [1].

Interestingly, the outpatients in this study receiving lithium maintenance therapy were those who received lithium during middle age: median (IQR) age onset of lithium therapy was 28 (21.0–41.5) years, and they received lithium for a short period: median (IQR) duration of lithium maintenance therapy was 2.97 (0.9–9.2) years. This may have resulted in a low incidence of CKD within this study. In a previous study, older age at the onset of lithium therapy was identified as an associated factor increasing the development of CKD [11]. The same study identified that CKD may develop within 5 years after starting therapy, and the odds of reaching CKD stage 3 were increased with a longer duration of lithium exposure [1]. On the other hand, it may be possible that the administration of lithium in young patients, or given over a short period, may be considered safe for patients. Therefore, to decrease the occurrence of CKD among those who are on lithium in maintenance therapy, renal function should be assessed at initiation and regularly monitored at least every 3–6 months; especially among those of an older age at the onset of lithium therapy, and having a duration of lithium therapy of over ten years [12]. Moreover, outpatients receiving lithium maintenance therapy, and advancing in age should also be especially careful in concerns to renal side effects.

In this study, despite the recommendations to check renal functions and lithium levels annually being in place, there were 154 outpatients with no reported levels of lithium, and one of these outpatients was found to develop CKD. Ninety-eight patients were not monitored for renal functions after starting the medication. It reflects the failure to meet the standard care for outpatients receiving lithium therapy. Therefore, to reduce the risk or enhance early detection of lithium-induced CKD, physicians should pay close attention and strictly follow the protocol for lithium usage. Although there were no obvious mechanisms of lithium-induced CKD, the earlier reports found cases of patients with chronic tubulointerstitial nephropathy on renal biopsy attributed to lithium, which led to progressive CKD over many years [13]. A recent meta-analysis suggested that the decline in GFR may be confounded by multiple other variables, such as medical comorbidities [14]. Some previous research suggested that a once-daily dose of lithium, independent of whether the preparation is sustained release or immediate release, may confer a lower risk of renal insufficiency compared to regimes with multiple dosing requirements per day [11]. Therefore, these issues should also be focused on in future research.

Concerning the history of psychiatric hospitalization, lithium intoxication, and the presence of CKD, the results of this study were similar to prior studies that found prior episodes of lithium intoxication could impact the rate or severity of kidney impairments [9]. Therefore, close observation of the side effects of lithium; such as hand tremors, and regular monitoring of serum levels of lithium may be necessary to prevent CKD in patients receiving lithium maintenance therapy. Additionally, psychoeducation regarding the risks of lithium intoxication; including dehydration and the use of nephrotoxic agents, should be provided to both patients and their families.

In regard to the presence of physical illnesses, as in non-communicable diseases; such as hypertension, dyslipidemia, and diabetes mellitus, this study found that five out of six outpatients receiving lithium and that developed CKD had had underlying non-communicable diseases. It may be possible that hypertension, dyslipidemia, and diabetes mellitus may have some association with the development of CKD, which should be investigated further. Prior studies have found complications of uncontrolled non-communicable diseases that lead to damaged kidneys and the development of CKD [6, 15]. The mechanism of hypertension impacts the kidney, by increasing intra-glomerular pressure, microvascular disruption, and inflammation [16]. Additionally, diabetes mellitus can also cause CKD, due to uncontrolled Hemoglobin A1c (HbA1c) levels disrupting the metabolism pathways in glycolysis and oxidative phosphorylation [17]. Dyslipidemia and low High-Density Lipoprotein (HDL) can lower kidney function and increase the risk of albuminuria. HDL has antioxidant, anti-inflammatory, and protective effects, which means a low HDL will reduce its protective function [18]. Moreover, triglyceride elevation can cause microvascular complications; however, its complete mechanism can still not be fully explained [19]. Therefore, outpatients receiving lithium maintenance therapy combined with the development of a physical illness should be closely monitored for CKD.

Although this was the first study investigating the incidence of lithium-induced abnormal renal function or CKD in Thailand, it had the limitation of being conducted retrospectively, with a patient sample size limited to only one university hospital; serving as a tertiary center in Southern Thailand. Although the sample size exceeded the required 178 patients, 307 patients making it large enough, the characteristics of the sample size might not be generalizable. Most of the outpatients were middle-aged and had a duration of lithium therapy of less than five years; hence, these results might not be applicable to all patients with a psychiatric illness receiving lithium maintenance therapy in Thailand. Therefore, further studies on lithium maintenance therapy-induced CKD should survey all ages and regions of Thailand. Unfortunately, we did not retrieve the data on the dates and duration the patients received nephrotoxic medications. Although all outpatients who developed CKD in this study did not have a history of the use of nephrotoxic agents, future and larger research should further explore the relationships between nephrotoxic agents and the development of lithium-induced CKD. Additionally, this study could not identify the factors associated with lithium-induced CKD, due to a limited number of outpatients developing CKD. Thus, future study needs to be replicated with a larger sample size population, be of a cohort design, and a multi-hospital study; including psychiatric hospitals.

## Conclusion

This study identified a low prevalence and incidence of lithium-induced CKD. Although lithium can be considered to be safe for maintenance therapy of psychiatric disorders, it is still warranted to include careful and regular monitoring. Especially, attention should be paid to older patients, those receiving lithium for a longer period, and those who present with comorbid physical illnesses.

## Data Availability

No datasets were generated or analysed during the current study.

## Declarations

We confirm that all methods were carried out in accordance with relevant guidelines and regulations.

## Abbreviations

CKD: Chronic kidney disease
DLP: Dyslipidemia
DM: Diabetes mellitus
HDL: High density lipoprotein
HT: Hypertension
